# Don’t Walk So Close to Me: Physical Distancing and Adult Physical Activity in Canada

**DOI:** 10.3389/fpsyg.2020.01895

**Published:** 2020-07-27

**Authors:** Katie M. Di Sebastiano, Tala Chulak-Bozzer, Leigh M. Vanderloo, Guy Faulkner

**Affiliations:** ^1^ Population and Physical Activity Lab, School of Kinesiology, University of British Columbia, Vancouver, BC, Canada; ^2^ ParticipACTION, Toronto, ON, Canada; ^3^ Child Health Evaluation Service, The Hospital for Sick Children, Toronto, ON, Canada

**Keywords:** COVID-19, pandemic, social distancing, moderate to vigorous physical activity, light physical activity, steps, incidental physical activity

## Abstract

**Background**: In response to the COVID-19 pandemic, physical distancing measures have been implemented globally. Canadians have been instructed to stay at home, which has likely resulted in significant changes in their physical activity. Using data from a national physical activity tracking app (PAC app), we aimed to determine device-measured physical activity levels immediately prior to and following the implementation of physical distancing measures in Canada to provide evidence for the development of physical activity recommendations for future pandemics or second wave infections.

**Methods**: Demographic and physical activity data were extracted from the ParticipACTION app (PAC app), using a 10-week (10 February to 19 April 2020) quasi-experimental design to determine changes in physical activity 4 weeks pre-pandemic and 6 weeks post-pandemic declaration. Weekly physical activity levels were monitored through wearable fitness trackers and health apps linked to the PAC app, to record moderate-to-vigorous physical activity (MVPA), light physical activity (LPA), and steps. Repeated measure ANOVA was used to determine changes over time (mean ± SE).

**Findings**: A total of 2,338 Canadians who were mostly 35–44 years old (26.6%) and female (90.2%) were included in the analysis. MVPA, LPA, and steps significantly declined immediately following the declaration of the pandemic (MVPA: pre-pandemic: 194.2 ± 5.2 min, post-pandemic: 176.7 ± 5.0 min, *p* < 0.001; LPA: pre-pandemic: 1,000.5 ± 17.0 min, post-pandemic: 874.1 ± 15.6 min, *p* < 0.001; steps: pre-pandemic: 48,625 ± 745 steps, post-pandemic: 43,395 ± 705 steps, *p* < 0.001). However, 6 weeks following pandemic declaration, MVPA (week 6: 204.4 ± 5.4 min, *p* = 0.498) had returned to pre-pandemic levels. LPA (week 6: 732.0 ± 14.3 min, *p* = < 0.001) and steps (week 6: 41,946 ± 763, *p* < 0.001) remained significantly lower than pre-pandemic levels at week 6.

**Interpretation**: Although MVPA returned to pre-pandemic levels, significant and sustained declines in incidental LPA and steps were observed. Attenuating the loss of incidental physical activity should be a public health priority in response to future pandemics or a second wave of a COVID-19 infection, as it may have significant long-term implications for the physical and mental health of Canadians.

## Introduction

On 11 March 2020 the World Health Organization (WHO) characterized the COVID-19 outbreak as a pandemic (World Health Organization, 2020). This has led to significant changes in daily life with specific recommendations and restrictions varying around the world. To inhibit the spread of COVID-19 in Canada, the Government of Canada (2020) has implemented physical distancing across the country. This has included the closure of schools, parks, playground facilities, trails, leisure facilities, and the introduction of physical distancing measures to keep people 2 m (6 ft) apart. During this time of physical distancing and instructions to “shelter in place,” it is intuitive to assume there will be consequences for children, youth, and adult physical activity participation and reaching the Canadian physical activity guidelines of 150 min of moderate-to-vigorous physical activity (MVPA) per week may be difficult (Canadian Society for Exercise Physiology, 2011). Initiatives to mitigate these consequences are necessary (Chen et al., 2020; Hongyan et al., 2020).

The British Association of Sport and Exercise Sciences (BASES) expert statement on physical activity and exercise during Covid-19 “Lockdowns” and “Restrictions” (British Association of Sport and Exercise Sciences, 2020) highlights a range of potential concerns as a result of lockdown procedures. Increases in sedentary behavior resulting from lost opportunities for incidental physical activity at school, work, and through active travel and exercise de-training, where the health benefits of previous physical activity are lost, may result. Finally, there may be consequences for mental health given the preventive role physical activity may play in protecting against such conditions (e.g., Mammen and Faulkner, 2013).

Physical activity may also have a more direct role in alleviating the consequences of the COVID-19 pandemic (Sallis and Pratt, 2020). For example, physical activity may target two key biological processes that react to infection – strengthening the immune system and reducing inflammation (Hojman, 2017). Physical activity is also effective in preventing and treating secondary conditions, such as heart disease and diabetes (Powell et al., 2018), that appear to be associated with increased risk of serious illness and death as a result of infection. As Sallis and Pratt (2020, p. 2) strongly conclude, “due to its multiple benefits, physical activity should not be an afterthought during this pandemic. Being active should be a key recommendation.”

Canadian public health guidance continues to recommend “going outside to exercise but staying close to home” while maintaining physical distancing from others (Government of Canada, 2020); however, surveillance of physical activity during physical distancing has been limited. Fitbit and Garmin, manufacturers of wearable fitness trackers, have released data collected from millions of device users around the world. During the week of 22 March 2020, Fitbit reported a decline in step count across every country examined compared to 2019, with European countries showing a more dramatic change ranging from a 7% (Germany) to 38% (Spain) decline in step counts. Canada saw a 14% decline in step count (Fitbit Staff, 2020). Garmin documented a worldwide decline in step count of ~500 steps/day from 15 March compared to 30 March 2020 and a transition to indoor activities such as virtual cycling (Garmin, 2020).

The data generated and methodological description from these company reports is extremely limited and the scientific rigor not reported, thus contextualizing physical activity recommendations based on this data is questionable. Differential effects may be observed in habitual or incidental physical activity versus purposeful exercise. Given the available sociocultural and physical supports, age, and gender may also influence physical activity participation. COVID-19 restrictions are unique by geographic area and effects on physical activity may not be uniform across Canada and globally. Recommendations may need to be tailored for different types of activity, sub-populations, or regions of Canada.

The purpose of this study was to investigate changes in the physical activity of Canadians 4 weeks prior to and 6 weeks following the implementation of physical distancing protocols in Canada, using data from a free and nationally promoted physical activity tracking app, the ParticipACTION app (PAC app). Findings will inform the development of tailored physical activity recommendations both at the current time but also in preparation for potential future restrictions as a result of a second wave of infections.

## Materials and Methods

### Study Design and Population

This was a 10-week quasi-experimental study examining changes in physical activity pre‐ and post-pandemic declaration (11 March 2020). The population was Canadian users (18+ years) of a free, publicly available physical activity tracking app – the PAC app. All of the 105,595 PAC app users were eligible for study inclusion. This secondary data analysis was reviewed and approved by the University of British Columbia Office of Research Ethics (#H20-01249).

### Data Collection

The PAC app is a nationally promoted physical activity app developed by ParticipACTION, a Canadian non-profit organization promoting physical activity. The PAC app delivers custom content, notifications, and weekly active minutes goals to each user based on their unique profile and rewards them with internal app awards (achievement badges) and external prizes (ballots for prize draws). The PAC app uses machine learning and continuous data collection processes collecting physical activity tracking, platform engagement, and user feedback. The app is unique in that it is a “national” app that is part of a broader social marketing strategy by ParticipACTION that includes strategic communications (“Everything gets better when you get active campaign”) and community challenges (“Community Better Challenge”). The continuous data collection process also allows for tracking the physical activity of Canadians who use the app.

Weekly summaries of app usage and individual physical activity were extracted from the internal app database. Data were extracted starting the week of 10 to 16 February 2020 – 4 weeks prior to the pandemic declaration – and continued until 13 to 19 April 2020 – 6 weeks following the pandemic declaration.

### Measures

Physical activity measures included objectively measured MVPA, light physical activity (LPA), and steps. Physical activity intensity was defined based on the device synced to the app. Currently, the PAC app is compatible and draws data from three manufacturers of wearable devices and their associated apps (Garmin, Fitbit, and Apple Watch), and two activity tracking apps with no associated wearable devices (Apple Health and Google Fit). For wearable devices, physical activity intensity was defined based on the device specific definitions, which typically use heart rate or step cadence. For example, a heart rate ≥60% heart rate maximum is classified as MVPA on Garmin devices. Heart rate is determined using the built-in monitors of the wearable device, and heart rate maximum was estimated using the equation: 220 minus age. When heart rate is not available, intensity is based on movement detection using the user’s smartphone built-in accelerometer, whereby MVPA was classified as a step rate ≥100 steps per minute and LPA as < 100 steps per minute. All activity data presented in the current study is recorded through a physical activity tracker (e.g., Fitbit) or app (e.g., Apple Health app) that is synced to the PAC app. To account for data syncing errors, limits were placed on the number of minutes of physical activity recorded per week (3,360 min for MVPA and 6,720 min for LPA). No restrictions were placed on step count.

Demographic information acquired during app registration was also extracted from the database. Such variables included age category, gender, and province or territory of residence.

### Statistical Analysis

All statistical analyses were completed using SPSS version 26.0. All app users with available data during the period of study (10 Feb to 19 Apr 2020) were included; however, preliminary examinations of the dataset revealed significant amounts of missing data in the sample (>50% of individual users did not have data on a given week). Additionally, the missing data were not missing at random as individuals with fewer minutes of activity were less likely to have complete datasets. As any attempts to impute missing data may introduce significant bias into the sample (Sterne et al., 2009), analyses were performed on complete cases only. A complete case was identified as an app user with physical activity data for all 10 weeks (10 February – 19 April 2020).

Demographic characteristics for complete cohort and completed cases were reported using frequency analysis and Pearson Chi-square analysis determined frequency differences between the age, gender, region, and physical activity levels groups. Activity data were reported as mean ± SE. Changes in physical activity over time (10 weeks) including MVPA, LPA, and steps were analyzed using one-way repeated measures ANOVAs with Bonferroni correction for multiple comparisons. Physical activity characteristics were also assessed over time by demographic and physical activity levels using mixed two-way repeated measures ANOVAs and reported using simple main effects with Bonferroni correction for multiple comparisons to assess interactions between the groups. Level of significance was set at *p* < 0.05 and effect sizes (*η* ^2^ *_p_*) were determined using partial eta-squared analysis. These effects can be interpreted as small (0.01), medium (0.06), or large (0.14; Cohen, 1988).

## Results

In the 10 weeks of the study, 23,173 Canadians logged on to the PAC app at least once and 2,338 (10.1%) had complete datasets. The majority of app users were 45–54 years old (26.7%), female (76.6%), and living in Ontario (34.7%). In the subset of users with complete data, the majority of users were 35–44 years old (26.6%, *p* < 0.001), female (90.2%, *p* < 0.001), and living in Ontario (34.0%, *p* = 0.096). Users with complete datasets were also more likely to meet the Canadian physical activity guidelines of ≥150 min of MVPA per week at week 1 (10 to 16 February 2020) compared to the whole cohort (complete data: 43.9% vs. whole cohort: 34.6%; *p* < 0.001) and are not considered sedentary (Tudor-Locke et al., 2012) by taking ≥5,000 steps per day (complete data: 83.4% vs. whole cohort: 80.3%; *p* < 0.001). Table 1 presents complete demographic characteristics of both cohorts.

**Table 1:** Demographic characteristics.

	Whole cohort (*n* = 23,173)		Complete data cohort (*n* = 2,338)	
	*n*	%	*n*	%
Age category				
18–24 years	990	4.3	70	3.0
25–34 years	3,425	14.8	317	13.6
35–44 years	5,422	23.4	622	26.6
45–54 years	6,185	26.7	599	25.6
55–64 years	5,192	22.4	540	23.1
65 + years	1,680	7.3	186	8.0
Unknown (18+)	277	1.2	4	0.2
Gender				
Female	17,749	76.6	2,109	90.2
Male	5,177	22.3	229	9.8
Other	245	1.1	0	0.0
Regions				
Atlantic Canada	2,330	10.1	263	11.3
British Columbia	4,090	17.7	422	18.2
Ontario	8,010	34.7	788	34.0
Quebec	3,669	15.9	332	14.3
The North	110	0.5	11	0.5
The Prairies	4,843	21	503	21.7
Unknown	119	0.5	19	0.8
Physical activity levels – week 110,41444.9				
≥150 min MVPA per week	3,602	34.6	1,027	43.9
<150 min MVPA per week	6,812	65.4	1,311	56.1
≥5,000 Steps per day	8,359	80.3	1,950	83.4
<5,000 Steps per day	2,055	19.7	388	16.6

### Changes in Physical Activity During the Pandemic

**Figure 1:**
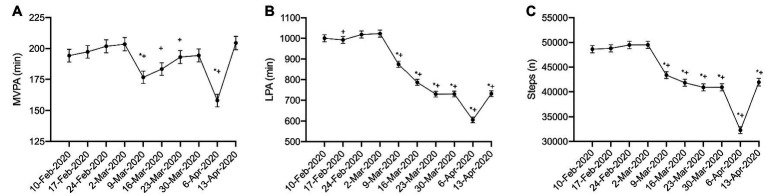
Physical activity during physical distancing. **(A)** moderate-to-vigorous physical activity (MVPA); **(B)** light physical activity (LPA); **(C)** Steps; (*) represented significant difference from 10-Feb-2020; (+) represented significant difference from 2-Mar-2020. For clarity, only selected significant differences are indicated on the figure.

As expected, there were significant differences over time for MVPA (*η* ^2^ *_p_* = 0.009; *p* < 0.001), LPA (*η* ^2^ *_p_* = 0.119; *p* < 0.001), and steps (*η* ^2^ *_p_* = 0.069; p < 0.001) in the sub-sample. In the 4 weeks prior to the pandemic, 10 February to 2 March 2020, MVPA remained relatively constant, with no significant difference during this time (Figure 1A; *p* = 0.208–1.00). MVPA significantly declined the week the pandemic was declared (9 Mar 2020) compared to the 4 weeks prior to the pandemic (Figure 1A; *p* < 0.001). This was followed by a further decline in MVPA during the week of 6 April 2020 (Figure 1A; *p* < 0.001); however, MVPA returned to pre-pandemic levels by 13 April 2020 (Figure 1A; *p* = 0.498–1.00). LPA (Figure 1B) and steps (Figure 1C) were also relatively stable prior to 9 March 2020 (*p* = 0.149–1.00); however, significant declines in both were observed beginning the week of 9 March 2020 (Figures 1B,C; *p* < 0.001). Further declines in LPA and steps were observed during the week of 6 April 2020; however, by 13 April 2020, these levels returned to the levels of activity observed after the implementation of physical distancing (23 March to 5 April 2020), though these activities remained lower than pre-pandemic levels (10 February to 2 March 2020; Figures 1B,C; *p* < 0.001).

### Differences in Physical Activity by Activity Level at Week 1

**Figure 2:**
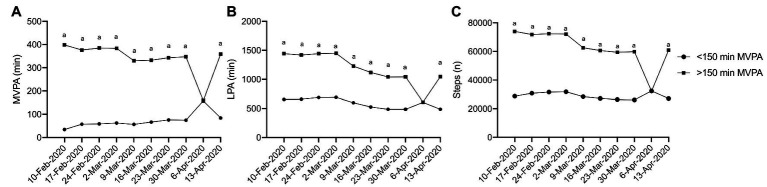
Physical activity during physical distancing week 1 activity level. **(A)** MVPA; **(B)** LPA; **(C)** Steps; (a) represents a significant difference between the two groups. For clarity, only selected significant differences are indicated on the figure.

Physical activity at week 1 (10 February 2020) of this investigation significantly impacted the amount of physical activity performed throughout the pandemic. App users who met the Canadian physical activity guidelines of ≥150 min of MVPA per week recorded significantly more MVPA (Figure 2A; *η* ^2^ *_p_* = 0.018, *p* < 0.001), LPA (Figure 2B: *η* ^2^ *_p_* = 0.144, *p* < 0.001), and steps (Figure 2C; *η* ^2^ *_p_* = 0.093, *p* < 0.0001) compared to app users who did not meet the Canadian physical activity guidelines. Interestingly, inactive app users (<150 min MVPA/week) demonstrated some resilience to declines of MVPA during the pandemic. Simple main effects analysis revealed increases in MVPA from the week of 10 February 2020 compared to the weeks of 17 to 24 February and 9 March, with further increases in MVPA observed during the remaining weeks of the pandemic (weeks of 16 March through 13 April 2020; *η* ^2^ *_p_* = 0.09, *p* = < 0.001). The MVPA of active adults (≥150 min of MVPA/week) followed the activity pattern of the whole cohort analysis. There were no observed differences in the pattern of response of LPA and steps between the active and inactive groups.

### Difference in Physical Activity by Age

**Figure 3:**
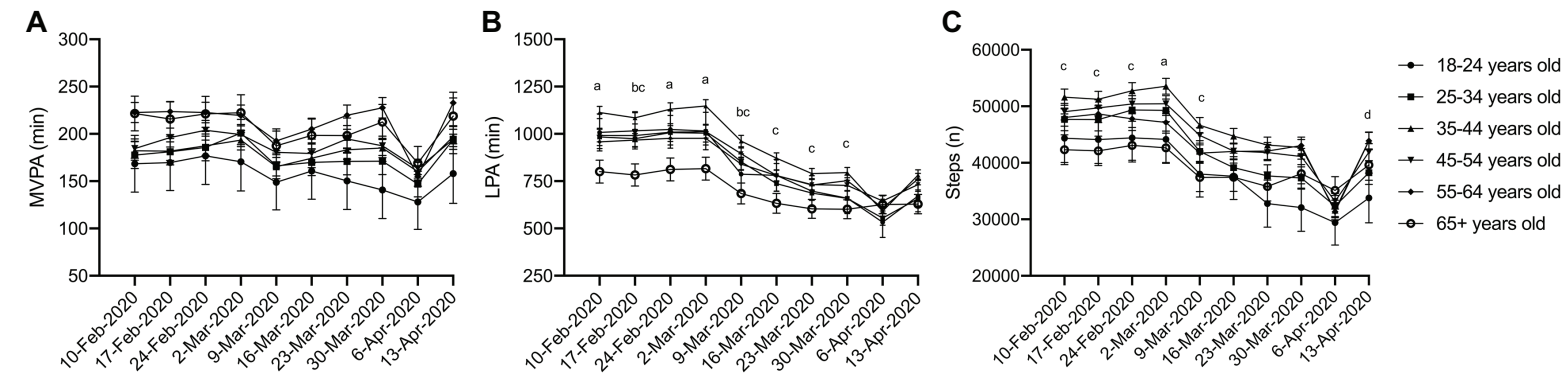
Physical activity during physical distancing by age. **(A)** MVPA; **(B)** LPA; **(C)** Steps; (a) 35–44 year olds are significantly different from 55 to 64 and 65+ year olds; (b) 65+ year olds are significantly different from 45 to 54 year olds; (c) 65+ year olds are significantly different from 35 to 44 year olds; (d) 55–64 year olds are significantly different from 25 to 34 year olds. For clarity, only selected significant differences are indicated on the figure.

No interactions were observed between age and physical activity over time for MVPA (Figure 3A; *η* ^2^ *_p_* = 0.003, *p* = 0.445). However, significant interactions, in that different age categories recorded different amounts of activity each week were observed in LPA (Figure 3B; *η* ^2^ *_p_* = 0.007, *p* < 0.001) and steps (Figure 3C; *η* ^2^ *_p_* = 0.006, *p* = 0.002), though these effects were small. Simple main effect analysis revealed that in general, older adults (55–64 and 65+ year olds) recorded less LPA and steps compared to younger adults (25–34, 35–44, and 45–55-year-olds). These findings persisted over the 10 weeks of data collection, which suggests that older adults recorded less incidental physical activity prior to the pandemic which continued into the pandemic.

### Differences in Physical Activity by Gender

**Figure 4:**
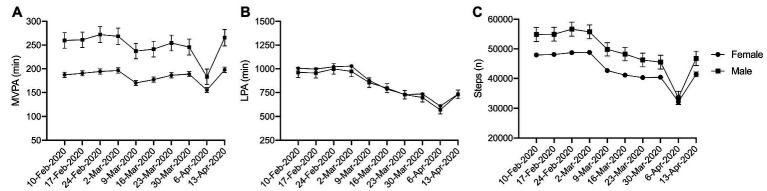
Physical activity during physical distancing by gender. **(A)** MVPA; **(B)** LPA; **(C)** Steps; No interactions observed.

No significant interactions were observed for gender (Figure 4; MVPA: *η* ^2^ *_p_* = 0.001, *p* = 0.154; LPA: *η* ^2^ *_p_* = 0.000, *p* = 0.624; steps: *η* ^2^ *_p_* = 0.001, *p* = 0.146), though a significant main effect was observed over time (MVPA: *η* ^2^ *_p_* = 0.006, *p* < 0.001; LPA: *η* ^2^ *_p_* = 0.044, *p* < 0.001; steps: *η* ^2^ *_p_* = 0.032, *p* < 0.001). Males accumulated more MVPA (Figure 4A) and steps (Figure 4C) than their female counterparts; however, a statistically significant difference was not detected, which may be due to the relatively small number of males with complete data.

### Differences in Physical Activity by Region

**Figure 5:**
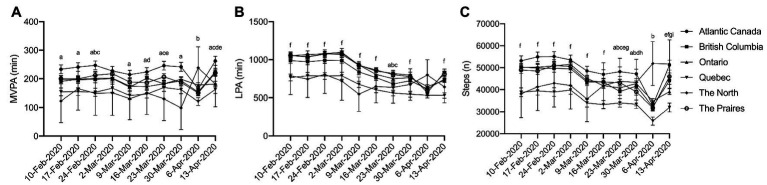
Physical activity during physical distancing by region. **(A)** MVPA; **(B)** LPA; **(C)** Steps; (a) Quebec is significantly different from Atlantic; (b) Quebec is significantly different from Ontario; (c) Quebec is significantly different from The Prairies; (d) Quebec is significantly different from British Columbia; (e) Atlantic Canada is significantly different from Ontario; (f) Quebec is significantly different from Atlantic Canada, British Columbia, Ontario, and The Prairies; (g) Atlantic Canada is significantly different from British Columbia; and (h) Atlantic Canada is significantly different from The Prairies. For clarity, only selected significant differences are indicated on the figure.

Significant interactions between regions of Canada and time were observed for all activity measures, in that people who live in different regions of Canada recorded different amounts of activity each week [Figure 5; (A) MVPA: *η* ^2^ *_p_* = 0.005, *p* = 0.001; (B) LPA: *η* ^2^ *_p_* = 0.006, *p* = 0.001; (C) steps: *η* ^2^ *_p_* = 0.007, *p* < 0.001], albeit these effects were small. Simple main effects analysis revealed that in general, Quebec recorded less MVPA (Figure 5A), LPA (Figure 5B), and steps (Figure 5C) compared to other regions in Canada. These finding persisted throughout the entire 10 weeks of the investigation suggesting that changes in physical activity due to the pandemic were not associated with region.

## Discussion

The purpose of this study was to investigate changes in the physical activity of Canadians immediately prior to and 6 weeks following the implementation of physical distancing protocols in Canada. Results indicate all measures of physical activity (MVPA, LPA, and steps) demonstrated a significant decline following the declaration of a global pandemic on 11 March 2020. Following the initial decline, MVPA had returned to pre-pandemic levels by 6 weeks (week of 13 April 2020) following the implementation of physical distancing protocols. The declines observed in LPA and steps were maintained in the 6 weeks following implementation of physical distancing protocols. This suggests that app users were able to successfully adjust their behavior to maintain their MVPA levels, but incidental physical activity (LPA and steps) has experienced a significant and sustained decline as a result of physical distancing. While significant differences were observed between app users in different age categories and regions of Canada, patterns of response to the pandemic appeared uniform for age, gender, and region. Notably, less active app users experienced minimal disruption in recorded MVPA over the 10 weeks of observation.

Additional declines in MVPA, LPA, and steps were observed during the 9th week of the study (6–12 April 2020). However, these additional declines returned to activity levels similar to week 8 (30 March 2020) by week 10 (13 April 2020). We hypothesize that these additional declines in physical activity may be attributed to the national statutory holiday that occurred over the 10 to 12 April 2020 weekend. App users may have significantly changed their behavior during the long weekend, which not only includes physical activity, but wearing fitness trackers and how they interact with the app during this time.

The existing literature examining physical activity during the COVID-19 pandemic is limited. To the best of our knowledge this is the first scientific research study to examine device-recorded physical activity during the COVID-19 pandemic and physical distancing. Fitbit and Garmin previously reported on changes in physical activity among users between 2019 and 2020. Fitbit reported a 14% decline in step count as a result of the pandemic in Canada (22 March 2019 vs. 2020). In comparison, we found a 12.8% decline in steps, a 14.6% decline in LPA, and a 13.1% decline in MVPA in the current study before and during the first week of physical distancing (Week of 2 March vs. 11 March 2020). These declines align with changes in physical activity of other North American countries, with the United States and Mexico demonstrating a 12 and 13% decline in steps, respectively (Fitbit Staff, 2020).

Garmin suggests that people are exchanging their typical physical activity with activities that can be done at home with minimal equipment. Skiing and golf have been replaced with virtual cycling and indoor workouts to maintain and exceed physical activity levels of prior to the pandemic (Garmin, 2020). These findings are reflected in the current study in that app users’ MVPA had returned to pre-pandemic levels by 6 weeks following the implementation of physical distancing. Similarly, Garmin also reported declines in step count of ~500 steps/day from 15 March compared to 30 March 2020, which is also reflected by the findings of the current study. Extending the Fitbit and Garmin reports, our results differentiate between step data and MVPA and demonstrate a rebound in MVPA that is not matched in LPA or steps.

Increased physical inactivity due to lost opportunities for incidental physical activity has been raised as a specific concern during physical distancing (British Association of Sport and Exercise Sciences, 2020). We observed this phenomenon in the current study when declines in LPA and step count were maintained in the 6 weeks of physical distancing while MVPA returned to pre-physical distancing levels over the same time period. This is both encouraging and concerning. It is encouraging in that physically active individuals were able to adapt and return to participation in MVPA 6 weeks after the start of physical distancing measures; however, incidental physical activity has likely been replaced with sedentary behavior. The suppression of LPA is concerning as higher levels of light-intensity and incidental physical activity have been independently positively associated with cardiorespiratory fitness (Ross and McGuire, 2011), and inversely associated with obesity (Fuzeki et al., 2017), blood glucose (Healy et al., 2007; Fuzeki et al., 2017), cardiometabolic risk biomarkers (Carson et al., 2013; Fuzeki et al., 2017), and all-cause mortality (Fuzeki et al., 2017; Loprinzi, 2017). This suggests that the observed declines in LPA of ~7 h per week may have significant implications for the health of Canadians.

Beyond the physical implications of declines in physical activity during the pandemic, potentially worsening mental health conditions are also a significant concern (e.g., Mammen and Faulkner, 2013). A review of the existing literature revealed 16–28% increases in anxiety and depression and an 8% increase in self-reported stress as a result of the COVID-19 pandemic (Rajkumar, 2020). Increased demand for mental health services during the pandemic and physical distancing may place further strain on limited mental health resources. Physical activity may reduce anxiety and depression and is recommended as a first line treatment for mild to moderate depression in Canada (Ravindran et al., 2016). Additionally, light-intensity exercise has been associated with increased well-being in older adults (Buman et al., 2010). The observed declines on physical activity in the current study may exacerbate the precarious mental health of Canadians during physical distancing.

### Study Implications

An important implication of this study is the need to explore how to attenuate reductions in LPA in particular in response to future pandemics or a second wave of COVID-19 infection. This may include concerted social marketing efforts to promote the importance to health of LPA, interrupting extended bouts of sedentary behavior and in clarifying the safety of physical activity outdoors while physical distancing. Our findings demonstrate the necessity for public health measures that provide extra space for everyone to engage in incidental activity through walking or cycling for example. This could include temporary reallocation of roadway space and keeping expansive green spaces open. Our finding that patterns of change appeared independent of age, gender, and region suggests such initiatives may be appropriate for many Canadians.

### Strengths and Limitations

Here, we used a large sample (>2000 participants) of device-measured physical activity data to reduce the bias associated with self-reported physical activity data. Self-reported physical activity overestimates the amount and intensity of physical activity of the respondent in question. Additionally, this study uses data from the PAC app, a continuously collecting physical activity tracking app. Consequently, we were able to document real-time changes in the physical activity of Canadians in response to the ongoing COVID-19 pandemic and physical distancing.

The PAC app collects data through linkages to physical activity tracking apps or other wearable physical activity monitors. These devices determine time spent in physical activity and intensity of activity using objective measures such heart rate and accelerometery. These devices reduce the bias associated with self-reported physical activity; however, each device uses its own proprietary algorithm to determine MVPA and LPA. Additionally, the use of these devices may have also changed as a result of physical distancing. For example, individuals who are now working from home may not carry their cellular phone around their homes, which would result in decreased steps and LPA recorded. Additionally, we observed a maintenance of MVPA in inactive Canadians during the 6 weeks of physical distancing. This maintenance might be explained by alterations in the way users interact with their fitness trackers and the PAC app. Motivated “active” individuals may adhere more to tracking their physical activity.

As described in the statistical analysis section of this manuscript, the data pull from the PAC app had significant amounts of missing data, and the missing data were not missing at random as individuals with fewer minutes of activity were less likely to have complete data sets. As a result, ~10% of app users had complete data and these users were more active than the whole cohort. Thus, the physical activity levels reported here are most likely above those of the average Canadian (Statistic Canada, 2019) and caution should be used extrapolating the data. While, the absolute values of activity may overestimate activity levels of Canadians, the trends of declining activity appear to reflect the whole cohort (data not shown). PAC app users also predominately identify as female (~75% of users in the whole cohort, ~90% of users with complete data). Therefore, generalization of the results for males in the current study is limited.

## Conclusions

Significant and sustained declines in incidental physical activity (LPA and steps) were observed, while MVPA returned to pre-pandemic levels by 6 weeks of physical distancing. Attenuating the loss of incidental activity should be considered a public health priority in response to future pandemics or a second wave of COVID-19 infection, as declines in incidental activity may have significant long-term implications for both the physical and mental health of Canadians.

## Data Availability Statement

The raw data supporting the conclusions of this article will be made available by the authors, without undue reservation.

## Ethics Statement

The studies involving human participants were reviewed and approved by the Behavioral Research Ethics Board University of British Columbia. Written informed consent for participation was not required for this study in accordance with national legislation and the institutional requirements.

## Author Contributions

KD and GF conceived and designed the study, designed the methods, and extracted the data, oversaw the analysis, and interpretation of data, and drafted and revised the article. TC-B and LV contributed to the analysis plan and reviewed the manuscript. All authors contributed to the article and approved the submitted version.

## Conflict of Interest

TC-B and LV are employed by ParticipACTION in the roles of Behavioral Insights Manager and Knowledge Translation Manager, respectively. Data in this study is from the ParticipACTION app activity tracker. GF is chair of the ParticipACTION Research Advisory Group (RAG). The RAG provides advice to ParticipACTION about the direction that should be pursued with respect to its research, evaluation, and knowledge translation. ParticipACTION provides meeting expenses for the RAG to meet but does not provide any additional compensation.

The remaining author declares that the research was conducted in the absence of any commercial or financial relationships that could be construed as a potential conflict of interest.
